# The β_3_-Adrenergic Receptor: Structure, Physiopathology of Disease, and Emerging Therapeutic Potential

**DOI:** 10.1155/2024/2005589

**Published:** 2024-11-28

**Authors:** Julius T. Dongdem, Axandrah E. Etornam, Solomon Beletaa, Issah Alidu, Hassan Kotey, Cletus A. Wezena

**Affiliations:** ^1^Department of Chemical Pathology, School of Medicine, University for Development Studies, Tamale, Northern Region, Ghana; ^2^Department of Biochemistry and Molecular Medicine, School of Medicine, University for Development Studies, Tamale, Northern Region, Ghana; ^3^Department of Microbiology, Faculty of Biosciences, University for Development Studies, Tamale, Northern Region, Ghana

**Keywords:** β_1_, β_2_, β_3_-adrenoceptors, adrenaline, cancer, cardiovascular disease, discovery, eye diseases, metabolic disorders, pathophysiology, pharmacology, therapeutics

## Abstract

The discovery and characterization of the signal cascades of the β-adrenergic receptors have made it possible to effectively target the receptors for drug development. β-Adrenergic receptors are a class A rhodopsin type of G protein-coupled receptors (GPCRs) that are stimulated mainly by catecholamines and therefore mediate diverse effects of the parasympathetic nervous system in eliciting “fight or flight” type responses. They are detectable in several human tissues where they control a plethora of physiological processes and therefore contribute to the pathogenesis of several disease conditions. Given the relevance of the β-adrenergic receptor as a molecular target for many pathological conditions, this comprehensive review aims at providing an in-depth exploration of the recent advancements in β_3_-adrenergic receptor research. More importantly, we delve into the prospects of the β_3_-adrenergic receptor as a therapeutic target across a variety of clinical domains.

## 1. Introduction

A plethora of biomarker identification and the development of therapeutic drugs have been made possible by the discovery and characterization of G protein-coupled receptors (GPCRs) most especially β-adrenergic receptors which are the best characterized with distinctive features that make them suitable as drug targets. GPCRs and GPCR-related genes constitute ∼17% of the total protein-coding genome and are considered the most important drug targets in medicine and physiology representing 12% of all drug targets [1, 2]. Over the past few decades, in-depth studies on receptor structure, subtypes, cellular localization, receptor activation, and canonical/noncanonical signaling cascades have made it possible to effectively use β_3_-adrenoceptors as potential targets for treating a variety of harmful conditions ranging from metabolic disorders to cardiovascular and pulmonary disorders. Adrenergic receptors also known as adrenoceptors constitute a rhodopsin class A type of GPCRs that stimulate the sympathetic nervous system (SNS) [3]. Agonists of adrenoceptors include catecholamines (e.g., epinephrine and norepinephrine) and some medications (e.g., β-blockers and α_1_ and β_3_ agonists) that are used for the treatment of high blood pressure and asthma among others [4, 5].

**Figure 1:**
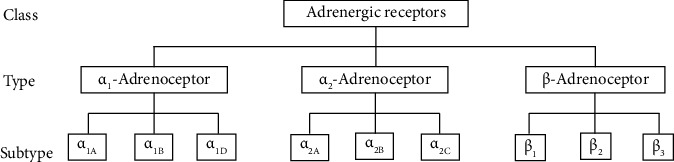
Classification of adrenergic receptors.

**Figure 2:**
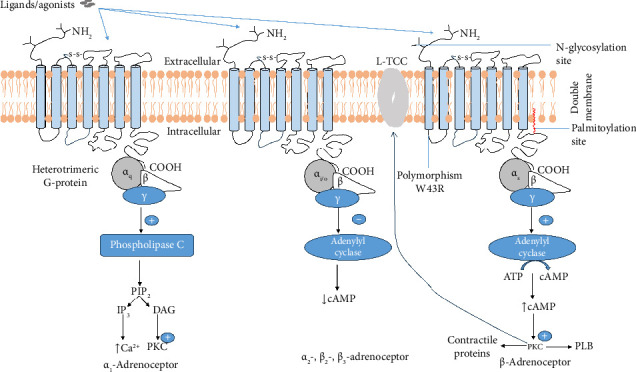
Major transduction mechanisms of the adrenoceptors. Different adrenoceptor types interact with different G protein types and therefore induce specific intracellular effects. Stimulation of an α_1_-adrenoceptor leads to conformational changes in the receptor that result in interaction with and subsequent dissociation of the G_αq_ subunit from the β*γ* subunits of the heterotrimeric G protein which in turn interacts with and activates phospholipase C (PLC) (left). Active PLC catalyzes the hydrolysis of phosphatidylinositol 4,5-bisphosphate (PIP_2_) into inositol 1,4,5-triphosphate (IP_3_) and diacylglycerol (DAG). Binding of ligands to β-adrenoceptors which are normally coupled to G_αs_ results in interaction with and subsequent activation of adenylyl cyclase (AC) (right panel). Active AC then synthesizes cyclic adenosine monophosphate (cAMP) from adenosine triphosphate (ATP). However, when α_2_-adrenoceptor is stimulated (middle panel), it is the G_αi/o_ subunit that dissociates from the β*γ* subunits to inhibit AC activity, thus leading to reduced intracellular cAMP concentration. Both β_2_- and β_3_-subtypes may also engage the G_αi/o_ protein.

The human genome contains 9 adrenoceptor genes that encode 9 different adrenoceptors of which 2 major groups (α- and β-) have been recognized. In 1948, they were grouped into 3 distinct families (i.e., α_1_-, α_2_-, and β-adrenoceptors) based on their pharmacological characteristics, sequence similarity, localization, and signaling mechanisms [3, 6]. More recently, each family has further been subdivided into 3 subtypes based on their affinities to adrenergic agonists and antagonists (Figure 1). The α_1_-adrenoceptor family is predominantly coupled to G_q_ proteins and has been subdivided into α_1A_, α_1B_, and α_1D_ while the α_2_-adrenoceptor family is usually coupled to G_i/o_ proteins and has likewise been subdivided into α_2A_, α_2B_, and α_2C_ subtypes (Figure 1) [7]. On the other hand, though β_1_-, β_2_-, and β_3_-adrenoceptors of the β-type (based on their affinities to adrenergic agonists and antagonists) are coupled to G_s_ proteins, β_2_- and β_3_-subtypes may also couple G_i/o_ proteins [8–12]. Ligand or agonist binding on adrenergic receptors therefore trigger intracellular cascade of events through direct interaction of a specific *α* subunit of the G protein (guanine nucleotide-binding protein) which is dependent on the particular receptor family (Figure 2). Each G protein comprises of 3 distinct subunits named α, β, and *γ* and is therefore a heterotrimeric complex.

Adrenoceptors are expressed by most cells of the human body. They are present in almost all peripheral tissues as well as several neurons of the central nervous system (CNS) [13]. Precisely, α_1A_ is predominantly expressed in the cerebral cortex, cerebellum, heart, liver, prostate, and lymphocytes (Table 1). The α_1B_ subtype is expressed in the spleen, kidney, endothelial cells, and osteoblasts and predominantly in the heart accounting for 70%–80% of the cardiac adrenoceptors, whereas α_1D_ is expressed in the cerebral cortex, carotid artery, aorta, lymphocytes, and heart [14–16]. The α_2_ subtypes are expressed in the brain, spleen, heart, kidney, and liver (Table 1). β_3_-adrenoceptors are predominantly found in brown adipose tissue, the gut, urinary bladder, and gallbladder. However, both β_1_- and β_2_-subtypes are located in the brain, lungs, liver, kidney, spleen, lymphocytes, and skin [16, 17]. Specific subtype activation therefore has a distinct function. For instance, the β_1_-subtype stimulation results in increased heart rate and cardiac contractility (Table 1). In addition, β_1_-subtype is reported to enhance cardiac dysfunction following myocardial infarction once over stimulated [18]. The β_2_-subtype regulates presynaptic norepinephrine release, vasodilation, ventricular function, and bronchodilation among others [19].

The β_3_-adrenoceptor is the most recently discovered of all adrenoceptor subtypes and has received less attention than both β_1_- and β_2_-adrenoceptors and therefore is the least studied so far among the β-subtypes [20]. To date, no literature has reported a crystal structure of the β_3_-adrenoceptor [21]. Perhaps, lack of the most suitable technology, lack of high affinity and high-avidity antibodies, animal and cellular models, and interspecies differences may explain this drought of information [22]; however, a substantial amount of literature over the past 3 decades has unraveled the β_3_ subfamily as an emerging target for novel pharmacological approaches in several clinical disciplines which has aroused a lot of interest among many scientists. Though not found in the Protein Data Bank (PDB), Nagiri et al. [23] recently reported a cryogenic electron microscopy structure of the β_3_-adrenoceptor bound G_s_ protein with agonist mirabegron revealing the molecular basis for β-adrenoceptor subtype selectivity that will allow the design of more selective drugs with fewer adverse effects. For instance, pharmacological agonists such as SR 58611, BRL 37344, and CGP 12177 activate the β_3_-adrenoceptor but have little effect on β_1_- and β_2_-adrenoceptors [24]. On the other hand, some agonists such as mirabegron (YM178) and solabegron (GW427353) are species-dependent and have higher affinity for the human β_3_‐adrenoceptor than that of rodent. Conversely, others such as BRL 27344, ritobegron (KUC-7483), and CL 316243 apparently have higher affinity for the rodent than the human β_3_‐adrenoceptor [20, 25]. Compared to both β_1_ and β_2_, β_3_‐adrenoceptor requires higher quantities of catecholamines to be stimulated [22].

β_3_‐adrenoceptors as forementioned relatively have a more restricted expression pattern in humans compared with β_1_‐ and β_2_‐adrenoceptors [26]. β_3_‐Adrenoceptors also have a unique ligand recognition profile for several antagonists including propranolol previously considered to inhibit all β‐adrenoceptor subtypes [27]. Besides, β_3_‐adrenoceptor agonists are currently preferred for development of therapeutics for metabolic diseases since norepinephrine and other nonselective derivatives have adverse side effects on cardiovascular tissue which have limited their use.

In this review, we provide a current insight on the structure, signaling, and functions of the β-adrenoceptors. We particularly explore the emerging potential of the β_3_-adrenoceptor as a versatile therapeutic target, comprehensively shedding more light on its implications for cancer therapeutics, metabolic disorders, neurological conditions, pulmonary disorder, asthma, and chronic obstructive pulmonary disease (COPD) among others.

## 2. Discovery of the β_3_-Adrenoceptor

**Figure 3:**
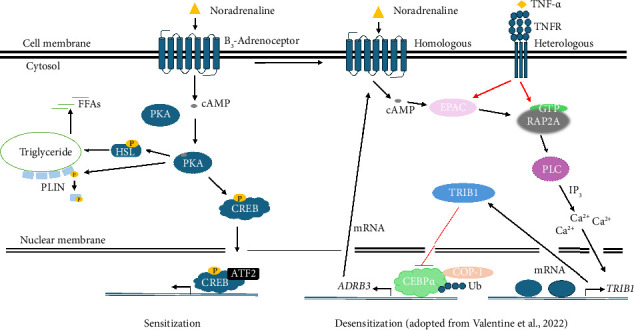
*β*_3_-Adrenoceptor signaling and desensitization. Stimulation of the *β*_3_-adrenergic receptor through catecholamine binding results in a rise in intracellular cAMP concentration which binds and activates PKA. Activated PKA phosphorylates and activates lipases (e.g., HSL, PLIN, and CGI-58) to increase adipocyte lipolysis releasing free fatty acids (FFAs) and glycerol. PKA also phosphorylates CREB which translocates into the nucleus. CREB and ATF-2 (also known as CRE; cAMP response element) together form a transcription factor that regulates expression of thermogenic genes. In homologous desensitization, cAMP also activates the EPAC/RAP pathway which is synergized by tumor necrosis factor receptor (TNFR) activation (i.e., heterologous desensitization) through TNF-*α* binding. Activated RAP2A in turn activates PLC which hydrolyzes PIP_2_ into IP_3_ and DAG. IP_3_'s action on endoplasmic reticulum increases intracellular Ca^2+^ concentration which induces transcription of *TRIB1* gene. Resulting TRIB1 protein recruits COP-1, an E3 Ub ligase whose activity leads to the degradation of CEBP*α* and subsequent downregulation of *ADRB3* gene expression.

The history of the discovery of the β-adrenoceptors is well documented by several scientists [28, 29]. Briefly, in the latter parts of the 1800s, John Newport Langley (1852–1925) first postulated the concept of specific receptors on the cell that bind drugs or substances and by so doing initiate biological effects or instead inhibit downstream cellular functions [30, 31]. Paul Ehrlich (1854–1915) about the same time observed that these receptors were selective [32]. In 1897, John Jacob Abel (1857–1938) successfully isolated an impure form of would be “epinephrine” from the adrenal gland of sheep that regulated blood pressure as an agonist on these receptors [17, 33, 34]. However, these concepts were not fully accepted by the scientific community until in 1948 when Alquist [6] clarified adrenaline's actions on 2 distinct receptors, the so called *α*- and β-receptors, and further established the concept that a single sympathetic mediator produced both excitatory and inhibitory responses. Lands and colleagues [35] in 1967 suggested subdividing the β-type adrenoceptors into 2 broad categories β_1_- and β_2_- based on differential order of potencies against 12 agonists and antagonists in several organ bath preparations [35]. Barely 5 years after this subclassification, scientists provided experimental evidence to demonstrate that some β‐adrenergic–like responses were not mediated by either β_1_ - or β_2_-adrenoceptors [36], such as lipolytic responses in rat white adipose tissue [37], smooth muscle relaxation responses in rat colon [38], and human urinary bladder [39], which looked to be mediated by β‐adrenoceptors based on the order of potency of catecholamines. It was not until 1984 when Arch [40] provided the first evidence for the existence of β_3_-adrenoceptors by demonstrating a new class of β-adrenoceptor ligands that selectively induced lipolysis in rat brown adipocytes. They showed that a series of β-adrenoceptor ligands (BRL 26830A, BRL 33725A, and BRL 35135A) had remarkable antiobesity, antidiabetic, and thermogenic effects on mice β_3_-adrenoceptor with severe obesity and diabetes rather than classical β-adrenoceptors. However, the existence of a third subtype, the β_3_‐adrenoceptor, only became fully accepted after it was cloned in 1989 by Emorine et al. [41]. Since its discovery, research over the past 3 decades has unraveled new insights relating the β_3_-adrenoceptor signaling pathway (Figure 3) and β_3_-adrenoceptor–mediated functions (Table 1). Recently, for instance, involvement of the β_3_-adrenoceptor in urine concentration mechanism, fat mass reduction, and inflammatory processes has been described giving more opportunities for new therapeutic applications, and hopefully more details will be discovered in the near future [42–45]. A proposed fourth subtype (β_4_-adrenoceptor) has remained controversial to date [46, 47].

## 3. Gene and Protein Structure

### 3.1. The β_3_-Adrenoceptor Gene

The β_3_-adrenoceptor gene (*ADRB3* or *BETA3AR*) is located on the short arm of chromosome 8 in humans (8p12–p11.2; cytogenetic location: 8p11.23). Besides humans, β_3_-adrenoceptor has also been identified in other mammalian species including the rat [48], mouse [49], bovine, sheep, goat [50], and dog [51]. The mouse and human β_3_-adrenoceptor genes are 81% identical with the highest homology of 94% within the transmembrane (TM) domains and lowest at the *C*-terminus and third intracellular loop [20]. The mRNA transcript-start site for both mouse and human *ADRB3* genes is between 150 and 200 nucleotides upstream of the ATG translation start codon. Compared with β_1_- and β_2_- which contain 1 exon each, the β_3_-adrenoceptor genes comprise 2 exons (Table 2). In humans, a large exon (1.4 kb) encodes the first 402 residues. The first 388 amino acid residues of the mouse β_3_-adrenoceptor are encoded by this exon [52]. In humans, *ADRB3* gene is made of 1 intron which is absent in both β_1_- and β_2_-adrenoceptors. The second exon (700 bp) in humans encodes the 6 *C*-terminus residues of the β_3_-subtype. In the mice, the second exon which is 68 bp encodes the 12 *C*-terminus residues while the third exon contains the β_3_-adrenoceptor mRNA 3′ untranslated region [52]. The rat ortholog was found to contain 3 exons and 2 introns [53] having an extra exon and intron over that of humans. As such, the human, mouse, and rat β_3_-adrenoceptor primary sequences differ markedly in length and essentially at the *C*-terminus region of the receptor [52]. Three subtypes of β_3_-adrenoceptors (A, B, and C forms) have been described resulting from an alternative splicing of the mRNA which arises from alternative promoters or polyadenylation signals in a tissue-dependent manner [40]. The primary sequence of form A consists of 396 amino acids, whereas forms B and C, respectively, contain additional 12 and 6 amino acids besides the *C*-terminus amino acid [49, 54]. The *ADRB3* gene product (the β_3_-adrenoceptor) belongs to a family of β-adrenergic receptors (Figure 1) that mediate catecholamine-induced signaling. The receptor is mainly located in the adipose tissue where it is involved in the regulation of lipolysis and thermogenesis. The structure and function of the receptor has now been widely characterized.

### 3.2. Protein Structure of the β_3_-Adrenoceptor

The β_3_-adrenoceptor is a 408 amino acid glycoprotein found in membranes of several cell types and tissues. The extracellular *N*-terminus of the β_3_-adrenoceptor, like β_1_- and β_2_-, is located outside the cell membrane and is glycosylated (Figure 2). It is required for ligand recognition and binding [55]. Characteristic of all members of the GPCR superfamily, adrenoceptors contain 7 TM helices (TM1-TM7) made of ∼22–28 hydrophobic amino acids which stretch across the cell membrane in a serpentine fashion to form a stable structure within the lipid bilayer. The structure therefore has 3 intracellular and 3 extracellular loops connecting the 7 TM domains. Ligands bind to the TM helices in the crevices formed by the helices. Specific amino acid residues in each helix interact with ligands, allowing for precise recognition and binding. This region (the binding pocket) is highly selective, thereby enabling the receptor to differentiate between ligands [55]. Ligand binding and subsequent activation of the receptor are essentially dependent on the formation of a disulfide bond between Cys^110^ and Cys^189^, respectively, located within the second and third extracellular loops [21, 24]. The intracellular loops connect the cytoplasmic ends of adjacent helices between TM helices. Conformational changes in these loops facilitate interaction with G protein subunits, resulting in stimulation of downstream intracellular signaling cascades upon ligand binding [55, 56].

The *C*-terminus of the receptor is intracellular. It is essential for receptor signaling and regulation [56]. Various proteins bind to the *C*-terminus, including G proteins and other signaling molecules, modulating receptor activity and downstream signaling events. Interactions at the *C*-terminus are required for the receptor to effectively transmit signals [55]. β-Adrenoceptors are coupled mostly to G_s_-protein (rarely to G_i_-protein) and stimulate activation of the effector enzyme (second messenger), AC (Figure 2). An initial computer modeling revealed an image of the ligand-binding site and β_3_ receptor-ligand interactions (source: https://www.uni-graz.at/%7Ebinder/science/b3adrenoceptors.html) [22]. Asp^117^ (within TM3) was identified to be involved in ligand binding via mutagenesis. Other amino acids implicated in ligand binding include Ser^169^ (TM4), Ser^209^, Ser^212^ (TM5), and Phe^309^ (TM6). TM2 and 7 are required for G_*α*s_ subunit binding as well as receptor activation possibly through Asp^83^ (TM2) and Tyr^336^ (TN7) [22, 56].

Like most GPCRs, the β-adrenoceptors are modified by Cys palmitoylation referred to as S-palmitoylation (Figure 2, right panel). Protein S-palmitoylation is a covalent and reversible lipid post-translational modification of a eukaryotic protein in which the lipid is specifically targeted for Cys residues of the protein. S-palmitoylation is catalyzed by palmitoyltransferases in mammals in which palmitate is invariably attached covalently to a specific Cys residue of a protein forming a thioester bond. The reverse reaction in which the lipid is removed from the protein is catalyzed by acyl-protein thioesterases. S-palmitoylation mostly occurs in membrane proteins such as cell surface receptor proteins. Essentially, S-palmitoylation increases hydrophobicity of proteins, thereby enhancing their membrane association. Additionally, S-palmitoylation is thought to enhance subcellular localization, protein–protein interactions, and binding capacities of a protein.

**Figure 4:**
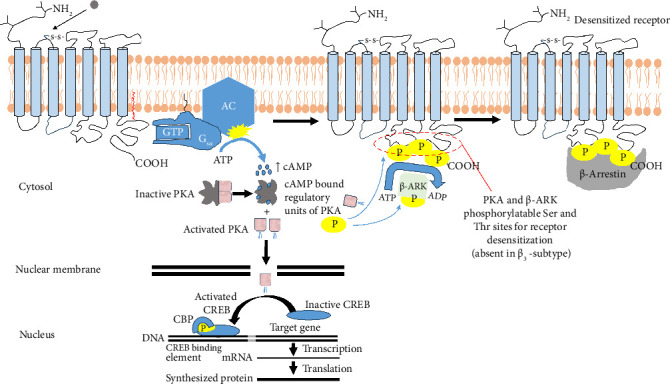
*β*-Adrenoceptor activation and regulation. When intracellular cAMP concentration rises resulting from receptor activation, cAMP molecules bind the regulatory subunits of PKA resulting in the release of active catalytic subunits of PKA which migrate into the nucleus where they catalyze phosphorylation of CREB. Once phosphorylated, CREB, a signal-regulated transcription factor, recruits a coactivator CBP which together induce gene transcription upon binding CRE. Activated PKA subunits may phosphorylate the receptor directly on Ser and Thr residues of intracellular loops or indirectly via phosphorylation of β-ARK. Phosphorylation activates β-ARK which in turn phosphorylates Ser and Thr residues of the β-adrenoceptor upon recognizing its stimulated configuration which decouples the Gs subunit from the receptor by steric exclusion. β-Arrestins bind the phosphorylated receptor. This prevents the receptor from further interaction with the G_s_ subunit of the trimeric G protein and acts as an adapter to initiate receptor endocytosis, subsequently sequestering and desensitizing the receptor (right panel).

Both human and mouse β-receptors are modified at the canonical S-palmitoylation site, i.e., Cys^358^ (located in the fourth intracellular loop) within the *C*-terminal tail; however, additional sites such as Cys^361^ in mouse and Cys^153, 292, 363^ in human β_3_-receptors which are differentially S-palmitoylated have recently been described [57]. Palmitoylation of Cys^361^ residue has been demonstrated to mediate ligand-induced activation of AC (Figure 4) [58]. The pharmacology of β-adrenoceptors has been extensively reviewed [20, 59–62]. Apart from the structural distinction between β_3_- and β_1_-/β_2_-receptors, β_3_'s pharmacological profile is also somewhat different. β_3_-Adrenoceptor shares 51% amino acid sequence similarity with the β_1_- and 46% similarity with β_2_-adrenoceptor, whereas β_1_ and β_2_ share 54% sequence homology (Table 2) [24, 63–65]. The main distinction between β_3_- and β_1_-/β_2_-adrenoceptors is that the third intracellular loop and the *C*-terminus domain of the β_3_-adrenoceptor lack phosphorylatable Ser and Thr sites for cAMP-dependent protein kinase A (PKA) and other GPCR kinases such as the β-receptor kinases (β-ARKs) present in β_1_/β_2_-adrenergic receptors (Table 2). PKA and β-ARK–dependent phosphorylation of these residues is required for rapid receptor desensitization and subsequent downregulation (Figure 4, Section 4.1) because it induces conformational changes at the *C*-terminal tail that prevents receptor-G protein binding and instead promotes β-arrestin binding of *C*-terminal tail of the receptor which triggers the internalization process [1]. To activate AC, the G protein must first interact with the *C*-terminal tail of the receptor which enables the G protein to exchange ADP for ATP with a consequential dissociation of the G_αs_ subunit from the *βγ* complex. Interaction of the G_αs_ subunit with AC activates AC. Activated AC then catalyzes the synthesis of cAMP from ATP which in turn activates several downstream cascades. Phosphorylation of receptor by PKA and β-ARK prevents this receptor-G protein coupling, thereby downregulating the receptor. This phenomenon makes the β_3_-adrenoceptor comparatively more resistant to agonist-induced desensitization [28, 64, 66, 67] as PKA and β-ARK–dependent phosphorylation of sites is absent in the receptor.

Interestingly, this characteristic, among others, renders the β_3_-adrenoceptor a unique therapeutic target for treatment of several chronic conditions [27, 68]. Structural homology between β_1_- and β_2_-receptors is 54% (Table 2) [24]. Structural homologies of β_3_-adrenoceptor of the human, bovine, monkey, hamster, guinea pig, rat, and mouse are considerably higher (∼80%–90%) [69]. These homologies are somewhat restricted to the TM domains, the membrane-bound proximal regions of the intracellular loops, and extracellular regions of the receptor responsible for ligand binding [63, 64]. β-Adrenoceptor signaling is mediated by AC activation via G proteins following ligand binding of receptor.

## 4. β-Adrenoceptor Signaling

The SNS is one of the 3 anatomically distinct divisions of the autonomic nervous system responsible for regulation of a plethora of homeostatic mechanisms including cardiac function (heart rate acceleration), gastrointestinal responses to food, urinary bladder contraction, thermoregulation, constriction of blood vessels, sweating, activation of goose bumps, pupil dilation (focusing), airway reactivity, glycogenolysis, and dilation of blood vessels in skeletal muscles [70–72]. Its main function is stimulation of the “fight-or-flight response” of the body. Catecholamines such as epinephrine (adrenaline) synthesized and released by the adrenal medulla, norepinephrine (noradrenaline) produced by sympathetic nerve endings (to a small extend) but majorly by the adrenal medulla, and dopamine synthesized in the substantia nigra, ventral tegmental area, and hypothalamus of the brain constitute the main stimulators of the SNS [73, 74]. These catecholamines circulate throughout the body and act on adrenergic receptors to elicit tissue-dependent responses.

When a specific catecholamine or agonist approaches a β-adrenoceptor, it interacts with the extracellular domain causing the receptor to activate [55]. The interaction allows the receptor to distinguish between different ligands, thereby initiating the specific corresponding signaling process [56, 75]. Ligand binding elicits complex changes in the conformation of the receptor resulting in receptor activation and subsequent initiation of the downstream intracellular signaling pathways (Figure 4). This change is pivotal in receptor activation because it exposes specific regions of the receptor downstream enabling them to likewise interact with a specific heterotrimeric G protein type within the intracellular compartment of the cell membrane. This interaction takes place at the intracellular loops as well as the *C*-terminus and is highly selective (Figure 4). β_3_-Adrenoceptor signals mostly through the activation of the G_αs_subunit which in turn dissociates from the G_β*γ*_ subunit complex and interacts with and activates AC. Active AC then catalyzes the conversion of ATP to cAMP leading to a cytosolic rise in cAMP concentration [75]. However, β_2_/β_3_-adrenoceptor may also engage the G_αi/o_ to downregulate AC activity and consequential reduction in cAMP levels.

G proteins (of which there are over 1000 types encoded in humans) are signaling molecules that transmit activation signals from receptors (i.e., GPCRs) to various intracellular effectors that ultimately result in a diverse range of cellular responses [54]. They are so called because they bind guanine nucleotides mainly guanosine diphosphate (GDP) and guanosine triphosphate (GTP) [76, 77]. As cytosolic cAMP levels rise, cAMP molecules bind the regulatory subunits of PKA leading to its activation and cAMP-dependent phosphorylation of gated ion channels [1, 78, 79]. Conformational changes in PKA due to cAMP binding result in the release of the regulatory subunits from PKA's catalytic subunits leading to its activation. Activated PKA catalytic subunits then migrate into the nucleus and phosphorylate CREB (cAMP response element-binding gene regulatory protein). Once phosphorylated, CREB recruits a coactivator CBP (CREB-binding protein) which together stimulate gene transcription upon binding CRE (CREB-binding element) (Figure 4).

### 4.1. β_3_-Adrenoceptor Desensitization

Receptor desensitization refers to a decreased responsiveness of a receptor following chronic or repeated exposure to an agonist and is a general characteristic of most GPCRs including α_2_- and β-adrenoceptors [80]. β_1_- and β_2_-adrenoceptors undergo classical β-arrestin–mediated desensitization (Figure 4), minutes after agonist stimulation to prevent hormonal overload [64]. Receptor desensitization is dependent on adrenoceptor phosphorylation by PKA, protein kinase C (PKC), or G protein linked receptor kinases (GRKs, β-ARKs) [1, 81]. Primarily, β-ARKs which recognize the agonist-occupied configuration of the receptors phosphorylate Ser and Thr residues of α_2_-, β_1_-, or β_2_-adrenoceptors. Once phosphorylated, the receptor binds an arrestin family of proteins with high affinity promoting uncoupling of the receptor from the stimulatory G protein (G_αs_). This prevents the receptor from further interaction with G_*α*s_ or serves as an adapter to couple receptor clathrin-coated pits to induce receptor-mediated endocytosis. These interactions ensure reduced receptor response and increased internalization and sequestration resulting from acute or chronic agonist exposure [73, 82–84].

Previous investigations in contrast demonstrated that β_3_-adrenoceptor fails to desensitize following acute exposure to agonists which is attributable to the lack of most of the structural determinants (i.e., the *C*-terminal β-arrestin–binding motif and the key phosphorylation sites) (Section 3.2, Table 2) that contribute to agonist-induced receptor internalization and consequent desensitization in α_2_-, β_1_-, and β_2_-adrenoceptors [63, 64, 66, 67, 85]. The β_3_-adrenoceptor, however, has been shown to desensitize only after long-term agonist-induced activation periods (hours to days) in some cell types which is suggestive of the involvement of cell- and/or species-specific mechanisms [86]. How then is the β_3_-adrenoceptor desensitized? What are the molecular alterations involved? Many studies have speculated about different desensitization mechanisms. Some have demonstrated a downregulation of β_3_‐adrenoceptor mRNA through decreased transcription of the *ADRB3* gene by exposure to some ligands such as tumor necrosis factor alpha (TNF-*α*) [28, 87–89]. However, whether reduced β_3_‐adrenoceptor mRNA synthesis necessarily translates into changes in the expression of the functional receptor protein remains controversial [28, 90]. This is because β_3_‐adrenoceptor–mediated function can diminish without any receptor protein downregulation suggesting that changes at the post‐receptor level may be contributing to receptor desensitization [91–94].

When stimulated, the β_3_‐adrenergic receptor interactions with G proteins subsequently result in a rise in intracellular cAMP concentration which bind and activate PKA (Figure 3). Phosphorylation of hormone-sensitive lipase (HSL) and perilipin (PLIN) by activated PKA increases lipolysis [94, 95] and also stimulates thermogenic gene (FGF21, IRF4, ZFP516, etc.) transcription via activation of the ATF-2 (activating transcription factor 2) and the CREB [96, 97].

Recently, Valentine et al. [85] provided a model for the signaling events that ultimately lead to both homologous (which is dependent on β_3_‐adrenoceptor activation) and heterologous (which is dependent on the inflammatory cytokine, TNF-*α*) desensitization of the β_3_‐adrenoceptor in adipocytes. They demonstrated that both homologous and heterologous signal cascades merge at the EPAC (exchange protein activated by cAMP)-RAP2A (Ras-related protein Rap-2a)-PI-PLC (phosphoinositide-phospholipase C) pathway to activate a cascade of transcriptional events which lead to the repression of the *ADRB3* gene expression through targeted degradation of CEBP*α* (CCAAT enhancer binding protein alpha) by Ca^2+^ dependent induction of TRIB1 (Tribbles pseudokinase 1) gene expression in adipocytes in response to TNF-*α* or high-fat diet (HFD) exposure. TRIB1 recruits COP-1 (constitutive photomorphogenesis protein 1), an E3 Ub ligase whose activity results in the degradation of CEBP*α* and subsequent downregulation of *ADRB3* gene expression [98] leading to catecholamine resistance of the receptor (Figure 3). Therefore, this pathway may be therapeutically relevant in the treatment of obesity and other related metabolic diseases [85, 99, 100].

## 5. Pathophysiology and Therapeutic Potential of the β-Adrenoceptor

β-Receptors are detectable in several human tissues. They mediate catecholamine signaling to regulate a surfeit of homeostatic functions of the SNS in the myocardium, retina, myometrium, adipose tissue, gallbladder, brain, urinary bladder, and blood vessels among others [20]. As such, β-adrenoceptors have become attractive drug targets. Agonists and antagonists of adrenergic receptors are being targeted for the treatment of a variety of pathologies including overactive bladder, heart failure, metabolic syndromes (obesity, type 2 diabetes, etc.), hypertension, glaucoma, hypotension, angina pectoris, asthma, different cancers (colon carcinoma, breast cancer, etc.), premature labor, cachexia, control of colon motility, anxiety, and depressive disorders. They are used as adjuncts to general anesthetics, as vasoconstrictors, bronchodilators, etc. In addition, they are targeted for opioid withdrawal, for relaxation of uterine smooth muscle, relief of allergic states (including anaphylaxis), and for CNS stimulation.

### 5.1. Targeting the β-Adrenoceptor in Urinary Disorders

Urinary disorders, encompassing the syndrome of overactive bladder (nocturia, urgency, with or without incontinence) including stress incontinence, significantly impact patients' quality of life. Generally, lower urinary tract dysfunctions increase in the elderly and postmenopausal populations and are attributable to hormone imbalances which may induce changes in bladder contractile and/or relaxing mechanisms. The bladder detrusor muscles relax during storage to accommodate rising volumes of urine to acceptable pressure while the neck of the bladder and urethra contract in order to resist involuntary urine leakage. However, during voluntary micturition (urine expulsion), the bladder neck and urethra muscles instead relax enabling the detrusor muscles of the bladder to contract in order to void urine without much resistance [101]. Detrusor muscle relaxation can be induced by stimulation of all 3 adrenergic receptor subtypes. However, previous studies identified the β_3_-adrenoceptor as the most important adrenoceptor in human detrusor muscle relaxation [102, 103]. Since then, researchers have focused on developing innovative therapies aimed at restoring normal urinary function [101]. Later, researchers demonstrated that sympathetic system–mediated relaxation of the urinary bladder [104, 105] is dependent on cAMP-mediated β_3_-adrenoceptor-G_s_ coupled signaling in mammals such as humans, monkeys, and dogs [106–108]. β_3_-Adrenoceptors which are expressed in the smooth muscles of the detrusor mediate muscle relaxation, thereby facilitating the filling capacity of the bladder [109, 110]. Location of β_3_-adrenoceptors, considerably the most prevalent of the 3 subtypes in the lower urinary tract and urethra, is strategic for regulation of the urothelium and prostate function [111, 112]. β_3_-Adrenoceptor–specific agonists used clinically today capitalize on this mechanism to improve overactive bladder conditions to prevent urinary incontinence [113]. As such, selective β_3_-adrenoceptor agonists, e.g., BRL 37344, CL 316243, FK175, or YM178, enhance bladder function by decreasing voiding frequency [114–116].

Similarly, Kullman et al. [115] assessed the ability of β_3_-adrenoceptor agonists to treat bladder dysfunction (i.e., voiding frequency, urgency, and incontinence). Using Sprague Dawley rat ovariectomy model, they demonstrated that depletion of ovarian hormone increased voiding frequency and reduced bladder volume by about 25% in awake rats and induced irregular cystometrograms in urethane-anesthetized rats even though RT-PCR results of bladder tissue indicated no significant difference in the expression patterns of all 3 β-subtypes compared with SHAM rats. Immunostaining showed localization of the β_3_-subtype in urothelium and detrusor muscle indicative of strategic localization for effective regulation of detrusor muscle relaxation. In addition, they confirmed the selectivity of BRL 37344, TAK-677, and FK175 to β_3_-subtype in CHOK1 cells overexpressing all 3 β-subtypes by assessing the relative potency of cAMP concentrations. They provided evidence that intravenous administration of 0.1–500 *μ*g/kg of the β_3_-adrenoceptor agonists in urethane-anesthetized rats decreased voiding frequency, bladder pressure, and amplitude of bladder contractions while 10^−2^–10^−4^ M reduced baseline tone as well as spontaneous contractions. Intraperitoneal administration of 5 mg/kg BRL 37344 or TAK-677 in awake rats reduced voiding frequency by as much as 40%–70%. The results therefore indicate that ovariectomy-induced bladder dysfunction (increased voiding and reduced bladder capacity) is not associated with β_3_-subtype expression pattern nor affects bladder inhibitory effects of the β_3_-subtype agonists and is confirmative of the potential of β_3_-subtype selective agonists to be developed for the treatment of hyperactive bladder disease in the elderly especially in postmenopausal populations.

So far, mirabegron has proved to be the most promising β_3_-adrenoceptor agonist over the past 30 years exhibiting good efficacy and tolerability and has currently successfully passed Phase III clinical trials in adults [117, 118]. Reports indicate that mirabegron has been authorized in Japan for the treatment of overactive bladder conditions [119]. Of interest also is TRK-380, another selective human β_3_-adrenoceptor agonist discovered 2 decades ago which induces isolated detrusor strip relaxation of several mammalian species [120]. TRK-380-induced relaxation of mammalian bladder strips is apparently solely mediated by β_3_-adrenoceptor and does have an effect on both resting and contractile responses. Additionally, TRK-380 has limited or no activity on β_1_ nor β_2_-subtypes compared with mirabegron and may have less cardiovascular effects without any major sight effects on urethral tone [68, 101]. BRL 37344 and CL 316243 which are species specific and therefore more efficient in rodents than in humans have been withdrawn [121, 122].

### 5.2. Targeting the β-Adrenoceptor in Endothelial Cell, Vascular Smooth Muscle Cell, and Cardiovascular Disease

Being essential regulators of vascular physiology, the β-adrenoceptors are involved in the pathology of such conditions as hypertension, atherosclerosis, and heart failures and are therefore potentially druggable for therapeutics in this regard. Generally, cAMP-mediated β-subtype signaling in smooth muscle cells often leads to muscle relaxation (i.e., vasodilation, bronchodilation (airways), uterine muscle relaxation, gastrointestinal tract relaxation, and neurotransmitter release) [123, 124]. Tissue specificity of β-subtype distribution is a major determinant controlling mechanical functions of the corresponding smooth muscle. The β_2_-subtype, for example, is abundantly expressed in airway smooth muscle. Increase in intracellular muscle cell cAMP concentration mediated by β-adrenoceptor stimulation due to binding of the receptor by a specific agonist is the established key trigger that leads to smooth muscle relaxation. Increased cellular cAMP level leads to activation of PKA. Active PKA then phosphorylates IP_3_ receptor of sarcoplasmic reticulum causing Ca^2+^-influx which in turn activates and opens Ca^2+^-dependent K^+^ channels such as the MaxiK (large-voltage dependant Ca^2+^-activated K^+^) channel. PKA and CaMKII (Ca^2+^ /calmodulin-dependent protein kinase II) also downregulate myosin light-chain kinase (MLCK; a Ser/Thr kinase that phosphorylates smooth muscle myosin on Ser19 resulting in smooth muscle contraction) by phosphoylating it on Ser1760, thereby, decreasing contractility and promoting smooth muscle relaxation [122, 125, 126]. Smooth muscle myosin lacks intrinsic myosin ATPase and therefore relies on posttranslational modification; i.e., phosphorylation on its Ser19 by MLCK to display activity [127]. MLCK is a Ca^2+^/CaM-dependent kinase and therefore is most simply activated by high intracellular Ca^2+^ ion concentration [127]. β-Adrenoceptor–stimulated smooth muscle relaxation without changes in cAMP concentration has also been reported. In airway smooth muscle, G_αs_ can directly interact with and activate MaxiK channels via β_2_-subtype stimulation. Gastrointestinal tract smooth muscle relaxation via cAMP-independent relaxation mechanism resulting from β_3_-adrenoceptor activation apparently leads to opening of delayed rectified K^+^ channels rather than the MaxiK channels [122] and are therefore druggable.

Cardiovascular diseases constitute a global leading cause of mortality and morbidity with a high 5-year mortality rate of 42.3%. Recent global statistics report that about 26 million adults suffer from heart failure [128, 129]. The main GPCR subtypes predominantly expressed in mammalian hearts are β_1_- and β_2_-adrenergic receptors. Cardiomyocytes, cardiac fibroblasts, endothelial, and vascular smooth muscle cells are the most abundant types of cells in the mammalian heart. Cardiomyocytes make up 30% of all heart cells and are the principal components of the myocardium (i.e., the muscle layer of the heart) that provides contractile force [130]. In humans, β_1_- and β_2_-adrenoceptors are the principal regulators of cardiovascular function, respectively, constituting ∼80% and ∼20% of all β-adrenoceptors in cardiomyocytes under physiological conditions [62, 131]. The β_2_-subtype is mainly expressed in cardiac fibroblasts, endothelial, and vascular smooth muscle cells. The stoichiometry of β_1_- and β_2_-adrenoceptors is reported to change to ∼60:40 during myocardial infarction, and a marked increase in G_i/o_ protein is primarily suggestive of a selective downregulation of β_1_- and a significant upregulation of β_2_-adrenergic gene expression [132]. For this reason, β-adrenoceptors play vital roles in the pathophysiology of heart disease and are therefore essential drug targets [133, 134].

Besides the canonical β-adrenoceptor agonists (catecholamines), some hormones and growth factors (Figures 3 and 4) such as vasopressin [135], insulin [136], TNF-*α* [137], and prostaglandin *E* [138], may also modulate the receptor function. Stimulated β_1_-adrenoceptor mediates accumulation of cAMP via G_*α*s_-AC interaction consequently leading to upregulation of PKA activity, which in turn phosphorylates intracellular signaling molecules (Ca^2+^ channels) responsible for intracellular Ca^2+^ regulation in cardiomyocytes including phospholamban (PLB), T- and L-type Ca^2+^ channel (T-/L-TCC), and ryanodine receptor (RyR) and other cation channels involved in depolarization and subsequent generation of action potentials (e.g., the delayed rectifier K^+^ channel IKs) as well as proteins involved in regulation of contractile machinery (e.g., cardiac troponin I and cardiac myosin-binding protein C) [139]. Binding of only cAMP activates cyclic nucleotide-gated Na^+^ channels causing Na^+^ influx leading to rapid depolarization. This creates action potentials at a faster rate than just opening of T- and L-TCC in order to produce an increase in inotropy (force of contraction), chronotropy (heart rate), dromotropy (rate of conduction), and lusitropy (relaxation of myocardium during diastole) essentially providing an increase in cardiac output [132, 140, 141].

Furthermore, binding of Ca^2+^ to RyR of cardiac sarcoplasmic reticulum allows release of more Ca^2+^ ions into the cytoplasm during an action potential resulting in an increased inotropism [72]. Overstimulation of the β_1_-adrenoceptor which engages only G_*α*s_ mediates cardiotoxic signals such as cardiomyocyte apoptosis, cardiac hypertrophy, and eventually heart failure [21]. During heart failure, excessive sympathetic stimulation through G_*α*s_ results in the activation of the cardiotoxic β_1_-adrenoceptor-CaMKII pathway in parallel with PKA. Both enzymes have many common Ca^2+^ ion homeostatic protein substrates and other substrates targeted at regulation of gene transcription. As such, β_1_-adrenoceptor blockade is one effective way of treating heart failure. In addition, β_2_-adrenoceptor–linked upregulation of GRK2 and G_*α*i_ is observed in heart failure. The β_2_-adrenoceptor couples dually to both G_αs_ and G_αi_ proteins. G_αi_ pathway on the contrary activates GRK2 phosphorylation of the receptor eventually leading to β-arrestin binding and subsequent receptor desensitization (Figure 4). Therefore, the G_αi_ pathway opposes the G_αs_ coupled β-adrenoceptor positive inotropism to reduce heart rate and myocardial contractility which is beneficial. This way the G_αi_ pathway exhibits antiapoptotic and cardioprotective properties [18, 142, 143].

β-Blockers constitute one of the most widely prescribed drugs for the treatment of cardiovascular disorders such as hypertension and myocardial infarction [25]. β-Blockers (e.g., carvedilol, bisoprolol, and metoprolol) have proven effective and have shown potential of reducing morbidity and mortality in myocardial infarction [144–146]. The primary function of β-blockers (as the name suggests) in the cardiovascular system is downregulation of β-receptor signaling to basal levels to prevent harmful overactivation of G_*α*s_ proteins in heart cells [131]. However, β-receptor oversuppression may cause constriction of blood vessels and bronchi which may be side effects in the use of β-blockers where there is a global inhibition of β_2_-adrenoceptors in other tissues [147]. The β-arrestin–pathway biased β-receptor agonist, carvedilol especially, preferentially stimulates β-arrestin–mediated pathways while exhibiting inverse agonism toward G_αs_ signaling [148]. Carvedilol-stimulated β-receptor triggers a wide range of signaling events such as microRNA processing, epidermal growth factor receptor (EGFR) transactivation, and extracellular regulated kinase (ERK) stimulation. β-Arrestin–mediated EGFR transactivation apparently has a cardioprotective effect as it regulates epithelial tissue development and homeostasis. This has been demonstrated in transgenic mice overexpressing a mutant β_1_-receptor that lacked GRK phosphorylation sites and so lacked ability to initiate β-arrestin–dependent β-receptor downregulation, in which case apoptosis and heart dilation were enhanced [134, 149]. Therefore, it seems that β-arrestin–dependent β-adrenergic receptor signaling is advantageous for the heart.

Recent reports suggest that the β_3_-adrenoceptor is cardioprotective as they are expressed in cardiac and pulmonary vascular tissues and are upregulated in cardiac diseases. Moreover, β_3_-adrenoceptors produce negative inotropic effects. Previous investigations had established that the canonical β-adrenoceptor pathway in cardiac function via Ca^2+^ influx is L-TCC phosphorylation mediated by PKA which is in turn activated by intracellular rise in cAMP. The effect of β_3_-adrenoceptor stimulation on cardiac L-TCC Ca^2+^ current which underlies the plateau phase of the action potential for contraction in left ventricular myocytes was investigated by Zhang et al. [150]. Myocytes obtained from isoproterenol-induced male Sprague Dawley rat heart failure were used to demonstrate that the β_3_-adrenoceptor agonist BRL 37344 causes a 21% dose-dependent decrease in L-TCC Ca^2+^ current under normal physiological conditions and even greater inhibition (31%) in a failing heart at the same dosage. While a similar inhibition pattern of L-TCC Ca^2+^ current was observed with nadolol (an antagonist of both β_1_- and β_2_-subtypes), this inhibitory action was abolished by L748337 (a highly selective antagonist of β_3_-adrenoceptor) but not nadolol [150]. This implies that stimulation of positive ionotropic response by BRL 37344 through β_1_- and β_2_-subtype activation of L-TCC is mediated mainly by the classical cAMP/PKA mechanistic pathway but was abolished following BRL 37344 superfusion due to β_3_-adrenoceptor stimulation and not by β_1_- or β_2_-subtype activation. When endothelial nitric oxide synthase (eNOS) was inhibited with L-NAME (L-N^G^-Nitro arginine methyl ester) in the presence of BRL 37344, the inhibitory effect on L-TCC was abrogated indicative of the involvement of the NO pathway in β_3_-adrenoceptor–mediated negative ionotropic response [150]. When myocytes were incubated with pertussis toxin, the inhibitory function of L-TCC was again abolished by BRL 37344 indicating that pertussis toxin might have inhibited ADP-ribosylation of G_i/oα_ protein subunit, thereby negatively regulating PKG and allowing L-TCC to function. Zhang et al. [150] then concluded that β_3_-adrenoceptor inhibits L-TCC function in both normal and heart failing myocytes, and that β_3_-adrenoceptor–mediated inhibition of myocyte L-TCC is aggravated during heart failure. They further concluded that the mechanism by which *β*_3_-adrenoceptor inhibits L-TCC function may involve the, pertussis sensitive G protein and partially, mediated by NOS-dependent pathway.

**Figure 5:**
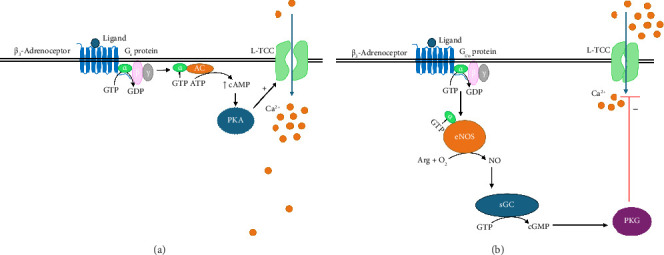
Mechanism by which *β*_3_-adrenoceptor mediates regulation of L-TCC Function. (a) Stimulation of L-TCC via cAMP/PKA pathway. (b) Inhibition of L-TCC via eNOS/PKA pathway. *β*_3_-Adrenoceptor activation may upregulate intracellular Ca^2+^ depolarization through positive regulation of L-TCC function to allow Ca^2+^ entry into myocytes (a). This is achieved via activation of the canonical pathway leading to intracellular increase in cAMP, subsequent activation of PKA, and phosphorylation of L-TCC by activated PKA. However, the *β*_3_-adrenoceptor is negatively coupled to eNOS apparently through G_αi/o_ subunit of the G protein (b). Interaction of G_αi/o_ with eNOS leads to activation of eNOS and synthesis of NO. NO then binds and activates sGC resulting in the synthesis of cGMP. PKG is activated upon binding cGMP and phosphorylates L-TCC to inhibit Ca^2+^ influx.

**Figure 6:**
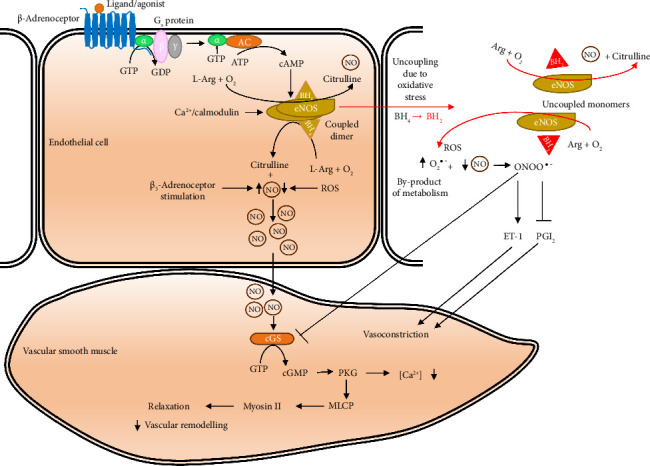
Mechanism of nitric oxide-mediated vasodilation and pathogenesis of endothelial dysfunction in vascular disease. In response to agonist/bradykinin/catecholamine stimulation of the β-adrenergic receptor which results in intracellular rise in cAMP and Ca^2+^ concentrations, cAMP and Ca^2+^/calmodulin bind and activate eNOS by disrupting its interaction with caveolin. Activated dimer of eNOS (coupled) then catalyzes the synthesis of NO by electron transfer through electron carriers to reduce O_2_. The critical cofactor, tetrahydrobiopterin (BH_4_), acts as the electron donor to reduce and activate O_2_ which enables oxidation of L-Arg to NO releasing citrulline as a by-product under physiological conditions. NO diffuses into the smooth muscle cells to activate cGS initiating a cascade of events leading to vascular smooth muscle relaxation. Uncoupling of eNOS results in the synthesis of O_2_^−^ a harmful free radical rather than antiatherosclerotic NO which is helpful. Uncoupling of eNOS is attributable to the oxidative depletion of BH_4_ to dihydrobiopterin (BH_2_), depletion of L-Arg (substrate), or accumulation of its analog (asymmetrical dimethylarginine) as well as eNOS S‐glutathionylation. Under oxidative stress conditions, BH_4_ is oxidized by O_2_^−^ and even more strongly by NOO− to BH_2_. NOO− is produced by scavenging of NO by O_2_^−^. Unfortunately, BH_2_ promotes functional dimeric eNOS uncoupling, thereby resulting in a vicious cycle of increasing harmful oxidants and oxidative stress. This phenomenon of excessive O_2_^−^ and oxidative stress leading to high ratio of BH_2_:BH_4_ is a major risk factor of cardiovascular disease pathophysiology.

It is now known that the β_3_-adrenoceptor is positively coupled to the G_αs_ subunit (in the human atrium, Figure 5(a)) which activates AC upon stimulation and therefore results in the synthesis of cAMP. Intracellular rise in cAMP results in binding of cAMP to PKA and activates PKA which in turn phosphorylates L-TCC. L-TCC opens to allow Ca^2+^ influx upon phosphorylation by PKA. On the other hand, the β_3_-adrenoceptor is negatively coupled to eNOS apparently through G_i/oα_ subunit of the G protein (in the human ventricle). G_i/oα_ subunit interacts with and activates eNOS which synthesizes NO from Arg (Figures 5(b) and 6). NO in turn binds and activates soluble guanylyl cyclase (sGC) resulting in the synthesis of cGMP. PKG is activated upon binding cGMP and phosphorylates L-TCC to inhibit Ca^2+^ influx [151, 152]. This pathway is cardioprotective and is pharmacologically relevant as this inhibitory ability may prevent overactivity of myocytes in heart failure. As such, the β_3_-adrenoceptor is of high interest for new therapeutic approaches for the treatment of heart failure.

Ferreira et al. [153] demonstrated that β_3_-adrenoceptor agonists attenuate myocardial remodeling in rat models. Similarly, results published by Kamiya et al. [154] suggest that chronic infusion of a β_3_-adrenoceptor agonist, BRL 37344, attenuates cardiac fibrosis and ameliorates diastolic dysfunction independent of blood pressure in an angiotensin II (Ang II)–induced hypertensive C57BL/6 J mouse model. Previous evidence had suggested an upregulation of β_3_-adrenoceptor expression in a heart that is failing in both man and animal models [155, 156]. Therefore, Kamayi and colleagues assessed the chronic effect of BRL 37344 in Ang II–induced cardiomyopathy mice and found that left ventricular end-diastolic pressure and end-diastolic pressure volume were significantly higher in Ang II–treated mice than controls; however, this increase was prevented in Ang II + BRL 37344–treated mice. Though heart rate was not different among the 3 groups (i.e., controls, Ang II only, and Ang II + BRL 37344), systolic blood pressure was significantly elevated in Ang II and Ang II + BRL 37344–treated mice. These results indicate improvement in myocardial stiffness induced by BRL 37344 treatment. Additionally, despite the fact that the left ventricular hypertrophy was stimulated in Ang II–treated mice and that BRL 37344 failed to prevent this, BRL 37344 however inhibited synthesis of collagen I/III, cardiac fibrosis, and lung congestion unlike Ang II only treated mice. This signifies cardioprotective effects of BRL 37344 which is thought to be associated with downregulation of transforming growth factor-*β*1 (TGFβ1) expression and subsequent phosphorylated SMAD2/3 mediated signaling [154]. Thus, chronic infusion of β_3_-adrenoceptor agonists may have potential therapeutic benefits for the treatment of heart failure.

### 5.3. Targeting β-Adrenoceptor in Arterial Hypertension

Together with their vasodilatory effects, β_3_-adrenoceptor agonists are a promising target in pulmonary arterial hypertension. Hypertension (i.e., high blood pressure) is a physical condition in which the blood vessels have persistently raised pressure to limits that can result in cardiovascular complications. Pulmonary artery hypertension is caused by endothelial dysfunction which is characterized by not only the inability of endothelial cells to vasodilate appropriately but also endothelial inflammatory activation. Hypertension stimulates proliferation of the vessel wall and narrowing of the lumen of the vessel. Persistent hypertension may result in lesions of the vessel walls which lead to vascular remodeling and ultimately development of atherosclerosis. In persistent hypertension, aneurysms (bulges) may occur in weakened vessels anywhere within the circulation, most especially around the aorta, and may result in vessel rupture posing a life-threatening situation for the patient [157]. Endothelial dysfunction is therefore associated with the development of several cardiovascular disorders such as heart failure and diabetes mellitus type 2. Current drugs used for treatment of pulmonary artery hypertension target dysfunctional endothelin (ET-1), NO, or prostacyclin (PGI_2_) pathways; however, overall survival with the disease remains poor (Figure 6) [158]. They most exclusively act as pulmonary vasodilators with poor safety profiles and so are inefficient.

The sympathoadrenal system regulates arterial blood pressure by controlling the vasculature, kidney, and heart rate [159]. Increased catecholamine secretion is reported to contribute to the pathogenesis of hypertension [160] suggesting that β-adrenoceptors may play a key role in the pathogenesis and maintenance of hypertension. Current knowledge on the molecular and biochemical basis of pathogenesis indicates a decline of the vascular β_2_-adrenoceptor signaling which leads to an impairment in vessel vasorelaxation while maintaining normal vasocontraction. This phenomenon is age-related. The underlying dysfunction is a consequential reduction in β-adrenoceptor–mediated cAMP synthesis in response to agonist activation and as such is associated with conditions involving altered cAMP synthesis such as hypertension, arterial insufficiency, orthostatic hypotension, and atherosclerosis [161, 162]. To date, no change in any specific factor can explain the impairment in β-adrenoceptor–mediated signaling suggesting that it may be as a result of multiple factors. Apparently, this impairment is linked to upregulated overexpression and activity of GRK2—the enzyme responsible for phosphorylation and subsequent desensitization of β-adrenoceptors; however, this mechanism is not fully understood [163].

Catecholamines and β-adrenoceptors contribute to the synthesis of NO. NO is synthesized by eNOS (a Ca^2+^/calmodulin-dependent enzyme) using BH_4_ as a cofactor. NO initiates a cascade of events that lead to smooth muscle cell relaxation and blood vessel dilation (Figure 6). Once synthesized, NO diffuses through the vascular endothelium into vascular smooth muscle cells where it reacts with Fe bound to the active site of sGC. NO-Fe interaction activates sGC. Active sGC in turn synthesizes cyclic guanosine monophosphate (cGMP, a second messenger) from GTP. Protein kinases such as protein kinase G (PKG) are activated by binding cGMP. Active protein kinases phosphorylate Ca^2+^ channels resulting in their closure to prevent further Ca^2+^ ion entry into the cell. These channels also enhance Ca^2+^ transport back into intracellular stores (sarcoplasmic reticulum), resulting in a reduction in cytosolic Ca^2+^ concentration [164]. PKG phosphorylates Ser/Thr residues of myosin-light-chain phosphatase (MLCP) to activate MLCP whose major function is to dephosphorylate the regulatory light chain (RLC) of the motor protein myosin-II and so negatively regulates actomyosin-based contractility to relax vascular smooth muscle and reduce blood pressure (Figure 6). This notwithstanding, other pathways which are independent of Ca^2+^ but involve phosphorylation of Ser residues of eNOS by some protein kinases have also been identified. One of such pathways involves β_2_-adrenoceptor in which eNOS is activated by kinases such as PKA and protein kinase B (AKT/PKB) [1]. Other functions of NO include inhibition of platelet aggregation and regulation of gene transcription among others.

The major cause of endothelial dysfunction is decreased NO bioavailability. However, there is evidence to suggest that increased reactive oxygen species (ROS) bioavailability, increased consumption of NO by ROS, and oxidative stress also contribute to the pathogenesis of endothelial dysfunction which is a common hallmark of cardiovascular disease [1, 165, 166]. Diabetes, angiotensin II, congestive heart failure, dyslipidemia (hypercholesterolemia), organic nitrates, asymmetrical dimethylarginine, aging, and smoking increase vascular oxidative stress which triggers eNOS dysfunction by enhancing uncoupling of the active dimeric eNOS. As eNOS uncouples, it synthesizes predominantly superoxides (O_2_^−^) rather than NO, thereby increasing the amount of harmful free radicals which intensify cellular oxidative stress while reducing concentration of NO. The biochemical and molecular mechanisms underlying eNOS uncoupling have been reviewed in detail elsewhere [1, 167, 168]. Bioavailability of NO is dependent on the balance of factors leading to its synthesis and degradation. Reduced expression of eNOS or eNOS activity is the major cause of low intracellular NO. However, NO is also scavenged by O_2_^−^ which leads to decrease in NO's half-life in the vasculature implying that increased oxidative stress is implicated in the pathogenesis of cardiovascular disease [169]. More importantly, O_2_^−^ reacts with NO to produce peroxynitrite radical (ONOO^−^) which further aggravates oxidative stress situation and drives pathological changes. ONOO^−^ also strongly inhibits PGI_2_ signaling (Figure 6).

Being major downstream regulators of intracellular cAMP synthesis, the β-adrenoceptors have been targeted for the management of hypertension, angina, postmyocardial infarction risk, congestive heart failure, tremor, arrhythmias, and chronic obstructive pulmonary disorders [170, 171]. So far, nebivolol has demonstrated potential as a novel target for treatment of pulmonary artery hypertension in animal models [172]. For instance, Perros et al. [172] demonstrated that nebivolol (a β_2_- and β_3_-adrenoceptor agonist and a β_1_-antagonist) downregulates overexpression of growth factors and proinflammatory mediators leading to reduced vascular remodeling and inflammation. Nebivolol also stimulates endothelium-derived NO release leading to pulmonary vasodilation which attenuates the hemodynamic severity of pulmonary artery hypertension, reduces right ventricular hypertrophy, and removes ROS during oxidative stress [173–175]. Since increased catecholamine secretion is reported to contribute to pathogenesis of hypertension and that β-adrenoceptor function is depressed in hypertension as aforementioned, the success of nebivolol might be in part due to the activation of β-arrestin signaling [176–178]. The molecular basis for mechanism of nebivolol action is that it antagonizes the G_αs_-coupling of β_2_-subtype and stimulates GRK-mediated phosphorylation of the receptor, thereby promoting recruitment of β-arrestins and ERK1/2 phosphorylation leading to receptor desensitization (Figure 4) [179]. Vasodilation property of nebivolol is achieved by several mechanisms including NO release, Ca^2+^ influx blockade through L-TCC, and antioxidative effect among others [180]. The effect of nebivolol may be a resultant effect of β-adrenoceptor subtypes rather than a single specific subtype as metoprolol (a β_1_-adrenoceptor selective blocker) does not yield similar outcomes. Nevertheless, these features of β-blockers especially of the third generation suggest therapeutic potential with better safety profiles than conventional blockers in the treatment of heart failure and pulmonary artery hypertension.

### 5.4. Targeting the β-Adrenergic Receptor in Obesity and Related Metabolic Disorders

Obesity is defined as BMI > 30 kg/m^2^. Obesity increases the risk of developing related metabolic disorders such as type 2 diabetes mellitus, atherosclerosis, hypertension, coronary heart disease, and cancer. Unfortunately, obesity has become a pandemic affecting about a third of the world's population [181]. β-Adrenoceptors particularly the β_3_-subtype have been targeted for the treatment of obesity and type 2 diabetes mellitus [182]. Earlier investigations showed that β-adrenoceptor blockade counter regulates decreased plasma glucose concentration after insulin-induced hypoglycemia but does not interfere with other hormonal (e.g., cortisol, glucagon, and growth hormone) responses to hypoglycemia. Scientists showed that propranolol exhibited ability to prevent epinephrine from initiating peripheral insulin resistance by raising plasma glucose levels in humans [183]. This is because epinephrine increases glycogenolysis, through a cascade of phosphorylations initiated by β-adrenoceptor binding, inactivates glycogenesis, and activates HSL and phosphorylase kinase [184, 185]. Since then, the choice of β-blockers for treating obesity and type 2 diabetes has remained controversial emanating from various meta-analyses because of 3 major reasons including development of insulin resistance, masking hypoglycemia which is more characteristic of nonselective β-blockers compared with selective ones, and development of dyslipidemia. β-Blockers seem to increase plasma triglyceride levels but reduce HDL cholesterol levels [186].

Later, the idea to potentially use β_3_-adrenoceptor agonists (e.g., GP-12177) for the treatment of obesity and type 2 diabetes mellitus stemmed from β_3_-subtype's regulatory function on lipolysis. Brown adipocytes contain multiple mitochondria and exclusively express uncoupling protein 1 (UCP1) which essentially ameliorate energy usage through thermogenesis while white adipocytes store excess energy as fat. Therefore, brown tissue activation with concomitant beiging (i.e., conversion of white adipose tissue to beige fat) increases energy expenditure and is a potentially beneficial strategy for treatment of obesity and related metabolic diseases [187, 188]. Beiging can be activated by cold climate, exercise, or pharmacological activation of the β_3_-adrenoceptor [189]. Mainly expressed on the surfaces of brown and white adipocytes in mammals, the β_3_-adrenoceptor is principally accountable for activation of brown adipocytes as well as the induction of beiging in white adipose tissue under cold temperature or adrenoceptor agonist stimulation. In addition, β_3_-adrenoceptor promotes release of insulin and glucose uptake by cells besides thermogenesis which ultimately lead to reduction in body weight [190]. Dysfunction of *ADRB3* gene therefore could lead to insulin resistance and obesity [191]. β_3_-Adrenoceptor agonists such as mirabegron are potent activators of brown adipose tissue thermogenesis as well as white adipose tissue beiging in rodents [192, 193]. Mirabegron is already approved at 50 mg for treatment of overactive bladder disorders. However, to achieve significant thermogenic effect requires higher doses of the order of 200 mg which is accompanied by several adverse effects. This among others is attributable for inability to commercially develop β_3_-adrenoceptor agonists for treatment of obesity and metabolic syndromes at this stage [194, 195].

Several polymorphic variations of the *ADRB3* gene exist that have been shown to be associated with many disease pathologies. The most common variant is the Trp64Arg mutation. Some investigations suggest that β_3_-adrenoceptor polymorphism (Trp64Arg mutation) is associated with insulin resistance and body weight gain [196, 197] which can lead to obesity and type 2 diabetes. Similar investigations also suggest that various metabolic phenotypes (including increased BMI) are associated with this Trp64Arg mutation in the *ADRB3* gene [198–200]. However, even though some investigators have reported controversial outcomes [201–203], more recently, meta-analysis carried out by Wang et al. [204] is in consonance with earlier suggestions that Trp64Arg mutation in the *ADRB3* gene might actually cause insulin resistance. Wang and colleagues [204] analyzed data from 8 papers using 1586 subjects who had been examined and found that Trp64Arg variant had a positive correlation with insulin level and therefore concluded that there might be an association between Trp64Arg and insulin resistance. They further stated that this correlation may be affected by the type of blood sample, obesity, and ethnicity and agreed that dietary ingredients among others may affect the degree of insulin sensitivity [205]. As β_3_-adrenoceptor signaling is essential for activation of brown adipocyte thermogenesis and lipolysis of white adipose tissue, β_3_-adrenoceptor dysfunction due to this mutation may account for various metabolic diseases including insulin resistance and obesity leaving affected individuals prone to developing type 2 diabetes [182, 183]. As such, β_3_-adrenoceptor agonists have potential to be developed for the treatment of insulin resistance and related metabolic disorders.

Additionally, Chen et al. [206] recently evaluated the effect of corylin (a flavonoid extract from *Psoralea corylifolia* L.) on browning and obesity. Chen and colleagues showed that corylin induces increased overexpression of beige- or browning-specific marker genes (cited1, hox9, pgc1*α*, prdm16, and UCP1) in 3 T3 L1 adipocytes, white adipose tissues, and brown adipose tissues which ultimately increase the browning process with subsequential increase in lipolysis and thermogenesis. Using HFD and DIO (diet-induced obesity) male C57BL/6 mice, they further demonstrated that corylin strikingly reduces body weight and fat accumulation and increases insulin resistance, mitochondrial biogenesis, and β-oxidation. As the effect of corylin was abrogated by treatment with EX527, (a sirtuin 1 (SIRT1) inhibitor) and L-748337 (a β_3_-adrenoceptor antagonist), the molecular mechanisms employed by corylin to enhance browning and lipolysis of adipocytes most likely involves SIRT1 and β_3_-adrenoceptor, suggesting that corylin may be therapeutically relevant for the treatment of obesity with both SIRTI and β_3_-adrenoceptor dependent pathways playing a vital role. Previous evidence indicated that SIRT1 an NAD^+^-dependent protein deacetylase attenuates obesity, promotes fat mobilization, augments mitochondrial metabolism, regulates glucose metabolism, and also inhibits inflammatory response [206–208].

In a slightly different approach, assessment of adipose tissue oxygen consumption and type 2 deiodinase protein expression levels in male C57BL/6J mice after treatment with liraglutide and the β_3_-adrenoceptor agonist, CL 316243, revealed remarkable loss of body weight in the mice suggesting potential for combined therapeutic development in the treatment of obesity and type 2 diabetes [209]. Liraglutide is a long-acting agonist of glucagon-like peptide 1 (GLP-1) receptor and shares 97% sequence homology with native GLP-1. Liraglutide has over the past decade been approved as a lifestyle therapy in the management of obesity [210]. Weight loss resulting from liraglutide (GLP-1A) signaling is largely attributable to loss of appetite and energy consumption. Scientists have demonstrated that GLP-1/1A receptors in adipocytes may induce brown adipose tissue thermogenesis and browning of white adipose tissue [211, 212]. Additionally, Oliveira et al. [209] established that liraglutide not only increases interscapular brown adipose tissue oxygen consumption but also further exhibits additive effects by enhancing β_3_-adrenoceptor–induced oxygen consumption in interscapular brown adipose tissue as well as inguinal white adipose tissue in mice with concomitant increase in UCP1 and type 2 deiodinase expression. This implies that liraglutide complements effects of β_3_-adrenoceptor–induced thermogenesis and increases type 2 deiodinase activity in brown adipose tissue suggesting that activation of brown adipose tissue depot may result from intracellular thyroid hormone activation via deiodinase activity which upregulates adrenergic signaling in adipocytes through the thyroid hormone receptor alpha (TR*α*) [209, 213]. Moreover, TR*α*-mediated signaling also in turn increases expression of thermogenesis-related genes [214].

The effect of green tea (*Camellia sinensis*) extract (GT) on β_3_-adrenoceptor–mediated regulation of white and beige adipose tissue lipolysis was investigated in [215]. Earlier studies had established that administration of GT potentially increases energy expenditure, lipid mobilization, and fat burning with concomitant reduction of body weight in obese animal models [216, 217]. Sousa-Filho et al. [215] used wild type (WT) and β_3_-adrenoceptor knockout (β_3_KO) male mice fed with standard diet (SD) or HFD, in 6 groups, treated with or without 0.5 g/kg body weight of GT for 12 weeks (i.e., (WT + SD and β_3_KO + SD), (WT + HFD and β_3_KO + HFD), and (WT + HFD + GT and β_3_KO + HFD + GT)). Histological analysis showed that GT attenuated final body weight and body weight gain, whereas adiposity index was increased by HFD. Reduction in weight of epididymal white adipose tissue in obese mice treated with GT is probably attributable to increase in lipolytic genes including Prkacb (PKA), Pnpla2 (Atgl), and Lipe (HSL) mRNA levels observed in western blots as well as SIRT, Ppargc1a, and UCP1 associated with thermogenesis only in the presence of the β_3_-adrenoceptor. Plasma insulin resistance was improved as treatment with GT reduced fasting glycemia in WT mice but did not appreciably modulate fasting glycemia in β_3_KO mice. More importantly, GT attenuated plasma leptin levels induced by HFD in both WT and β_3_KO mice with concomitant rise in adiponectin concentrations in β_3_KO but not in WT. Plasma levels of total triglycerides, total cholesterol, aspartate amino transferase (AST), and serum alanine aminotransferase (ALT) were raised by HFD treatment. Apparently, GT treatment reduced total triglyceride and cholesterol content in both WT and β_3_KO; however, these parameters remained higher in β_3_KO mice compared with WT. RT-qPCR results showed that GT upregulated FGF21 (fibroblast growth factor 21) and FGFr1 (fibroblast growth factor receptor 1) mRNA levels in brown adipose tissue of GT-treated β_3_KO mice when compared with WT, likely because of the requirement of FGF21 in order to induce thermogenesis and energy expenditure through activation of brown adipose tissue. Increased lipolysis with reduced adipocyte size and increased browning of subcutaneous white adipose tissue were therefore found to be β_3_-adrenoceptor–dependent. Treatment with GT also increased brown adipose tissue mRNA levels of lipolytic (oxidative) genes ADRB3/UCP1 as well as energy expenditure. Additionally, increase in subcutaneous white adipose tissue expression of SIRT1, Ppargc1a, and UCP1 mRNA levels observed is indicative of the induction of the browning process. Altogether, the data suggest that GT employs β_3_-adrenoceptor pathway activation to achieve some therapeutic effects [215].

**Figure 7:**
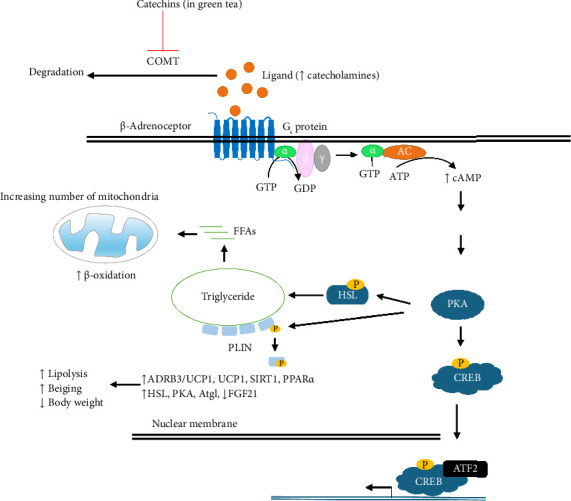
Hypothesized molecular mechanism by which green tea extract ameliorates weight loss. All β-adrenoceptors mediate catecholamine-stimulated lipolysis, beiging, and thermogenesis of adipocytes. Inhibition of COMT which degrades norepinephrine and epinephrine which are the natural ligands of β-adrenoceptors by catechins results in an increased concentration of ligands and therefore prolonged stimulation of the β-adrenoceptors especially of the β_3_-subtype. The resulting downstream cascade leads to upregulation of lipolytic genes Pnpla2 (Atgl) and Lipe (HSL), and thermogenesis-associated genes such as SIRT, Ppargc1a, and UCP1. The outcome is increased browning, energy expenditure, and fat oxidation of white adipocytes, thermogenesis in brown adipose tissues and loss in body weight. GT, therefore, employs majorly the β_3_-adrenoceptor pathway activation to achieve therapeutic effects.

The proposed molecular mechanism by which GT consumption may ameliorate body weight loss is by inhibition of the activity of catechol-O-methyltransferase (COMT) (Figure 7), one of the several enzymes that degrade catecholamines, catechol estrogens, and any substances containing a catechol structure through meta or para-O-methylation of OH^−^ groups of the catechols. The potent inhibitors of COMT present in GT are catechins. Catechins are flavan-3-ols (epicatechin, epicatechin gallate, epigallocatechin, and epigallocatechin-3-gallate) which are plant metabolites that exhibit antioxidant properties in plants. Catechins belong to a group of polyphenols known as flavonoids. Inhibition of COMT by catechins results in increased concentration of catecholamines (norepinephrine and epinephrine) available for stimulation of β-adrenoceptors and may thus lead to prolonged stimulation of the receptors. The resulting downstream events lead to increase in energy expenditure and fat oxidation in adipocytes which strategically contain high β_3_-adrenoceptors essentially leading to loss of body weight (Figure 7). This mechanism has been demonstrated *in vitro* [218]; however, more *in vivo* evidence is required to be conclusive. Nevertheless, this is indicative of therapeutic potential of catechins of which β-adrenoceptor–mediated pathways (particularly of the β_3_-subtype which are abundant on adipocytes) are targeted to achieve therapeutic benefits [215]. The β_3_-adrenoceptor is again therapeutically significant in this regard.

### 5.5. Targeting the β-Adrenoceptor in Vision, Ocular Tumors, and Other Eye Diseases

β-Adrenergic receptors are expressed in ocular structures (cornea, conjunctiva, lacrimal gland, uvea, trabecular meshwork, and retina) where they are presumed to play various pathophysiological functions. β-Adrenoceptors have already been identified as potential targets for the treatment of glaucoma, ocular neoplasms (e.g., hemangioma and uveal melanoma), etc. Though the role of β_2_-subtype in corneal epithelium regeneration is controversial at this stage [219], researchers have demonstrated that treatment with β_2_-subtype antagonists and consequential upregulation of kinase activity of ERK enhance corneal cell migration and corneal wound healing and may therefore have therapeutic potential [220, 221].

All 3 β-subtypes are expressed in human conjunctiva [222] and are thought to be involved in the pathogenesis of some conjunctival diseases, e.g., conjunctival inflammation [140]. Liu et al. [223] observed an irregular expression pattern of the β_1_-adrenoceptor in all epithelial layers of conjunctival biopsy specimens of patients with vernal keratoconjunctivitis. This indicates that the receptor might play a role in pathogenesis and, if so, may have therapeutic potential. Additionally, salbutamol and terbutaline (β_2_-subtype agonists) seemingly attenuate microvascular permeability as well as exhibit anti-inflammatory effects in allergic conjunctivitis. As such, β-adrenoceptors may therefore have potential for the treatment of allergic conjunctivitis [140, 224, 225]. Studies also indicate that β-adrenoceptors are involved in the regulation of tear secretion in lacrimal gland and may play an important role in the pathophysiology of dry eye disease [226, 227]. As such, manipulation of β-adrenoceptor pathways may be potentially helpful in the treatment of dry eye disease [228].

Researchers have demonstrated abundant expression of β_1_- and β_2_-adrenoceptors in both ciliary body and epithelium of the ciliary process in humans and other mammalian species [229]. Agonists of β-adrenoceptors were earlier reported to induce desensitization of the β-adrenoceptor-AC complex indicating that this may have a delayed effect on intraocular pressure reduction following application of topical agonists of β-adrenoceptors. However, both agonists and antagonists lower intraocular pressure [230]. Uveal vasculature vasoconstriction coupled with reduction in aqueous humor production might be another mechanistic explanation of intraocular pressure decreasing property of β-blockers. As some non-subtype selective β-adrenoceptor antagonists (e.g., timolol) and some β_2_-type selective agonists to other β-receptors in ciliary processes possess potent characteristic lowering of intraocular pressure in various mammalian species, β_2_-adrenoceptors may be pharmacologically relevant for the treatment of glaucoma [231–233]. Timolol is also reported to increase choroidal vascular tone. This is indicative of the involvement of the sympathetic system in the maintenance of choroidal vasculature [234, 235]. Researchers have further shown that the β_3_-subtype plays a role in choroidal cell invasion and elongation using BRL 37344 (a β_3_-type specific agonist) [236]. Additionally, β_2_-type in turn plays a role in the regulation of VEGF (vascular endothelial growth factor) and interleukin 6 (IL-6) expression in choroidal endothelium. This indicates that blocking these β-adrenoceptors will synergistically attenuate choroidal neovascularization which may leak and cause vision loss [237].

Expression, cellular distribution, functions, and therapeutic implications of the β-adrenoceptors in the retina have been reviewed by several scientists [140, 238–240]. All 3 subtypes of β-adrenoceptors have been isolated in the retina of some mammalian species including man [241, 242]. Based on receptor localization in retinal blood vessels and neural retina, they are perceived to play vital roles in retinal vasculature and possess neuronal function [209, 239]. Catecholamine overstimulation of the cardiovascular system under stress conditions such as hypoxia has been reported in rat models [243, 244]. Catecholamines activate β-receptors which in turn induce overexpression of HIF-1*α* (hypoxia-inducible factor-1*α*) and VEGF which together activate proangiogenic cascades leading to the formation of pathogenic blood vessels (abnormal growth of blood vessels) in various retinal diseases including retinopathy of prematurity and diabetic retinopathy [239, 245]. More recently, scientists have demonstrated that treatment with propranolol a β-adrenoceptor blocker sufficiently inhibits hypoxia-induced overexpression of proangiogenic factors, VEGF and HIF-1*α*, and subsequent pathologic neovascularization in the retina of animal models [246]. This implies that β-adrenoceptor blockade may be protective against retinal angiogenesis [247]. Other studies have also shown that the β_3_-adrenoceptor antagonists, L-748337 and SR59230A, are both capable of downregulating hypoxia-induced VEGF expression and release in the retina [248]. In addition, the β_3_-adrenoceptor agonist, CL 316243, has been demonstrated to attenuate retinal damage after N-methyl-D-aspartate (NMDA; a mimic of glutamate) injection [249]. These properties of the β_3_-subtype are pharmacologically relevant for the treatment of ischemic retinal diseases [239]. Jiang et al. [250] reported a novel β-adrenoceptor agonist (compound 49b) which is capable of attenuating VEGF levels in type I diabetic rat model suggesting that it may be protective against edema. The possible mechanism by which compound 49b achieves reduction of VEGF levels may be by increasing levels of insulin-like growth factor binding protein 3 (IGFBP-3) in diabetic retina via downregulation of eNOS and PKC signaling [250]. As some agonists and antagonists of β-adrenoceptors cause reduction of VEGF and are therefore antiangiogenic, these effects are likely regulated by different mechanisms, and it is important to fully understand these differential mechanisms.

An infantile hemangioma is a benign vascular neoplasm resulting from an abnormal proliferation of endothelial cells and enhanced angiogenesis [251]. Over the years, corticosteroids, vincristine, cyclophosphamide, and interferon alpha have been used in the treatment of infantile hemangiomas. Unfortunately, these drugs have multiple side effects [252–254]. Earlier studies showed that VEGF promotes proliferation of vascular endothelial cell and angiogenesis by binding VEGF receptor-2 on hemangioma derived endothelial cells [255–257]. Later, scientists observed that propranolol and other β-blockers caused regression of infantile hemangiomas which implies that β-adrenoceptors may play an important role in the pathogenesis of the disease [251, 258–261]. Propranolol, an antagonist of β-adrenoceptors, has therefore been targeted to treat infantile hemangiomas by employing its vasoconstriction and antiangiogenic properties and its ability to induce apoptosis since it downregulates intracellular cAMP concentration and consequently prevents PKA activation. Propranolol causes inhibition of eNOS and therefore no NO is released ultimately resulting in vasoconstriction [262, 263]. This implies that β-adrenoceptor antagonists and β-blockers have potential to be developed for treatment of hemangiomas.

Uveal melanoma is the primary intraocular malignant tumor arising largely from neoplastic melanocyte proliferation in the uveal tract. It is a relatively rare cancer and occurs in adult populations causing morbidity (blindness) and increasing risk of mortality. Current treatment includes tumor resection, transpupillary thermo-therapy, local radiation, or ultimately enucleation (surgical removal of the eye); however, ∼50% of patients eventually develop metastasis primarily to the liver which has an impact on patients' prognosis, giving 6 months survival on average [264, 265]. Recently, β-adrenoceptors have emerged as novel targets for inhibition of melanoma growth and dissemination [266]. The contribution of β-adrenoceptors in cancer progression (see Section 5.6) including melanoma has been established [267]. β_1_- and β_2_-subtype overexpression in uveal melanoma of all patients has been documented [266]. In addition, the role of the β_3_-subtype in melanoma growth and vascularization has been demonstrated in mouse models [268, 269]. Catecholamine levels are increased in cancer patients which tend to activate β-adrenoceptor signaling resulting in activation of eNOS and therefore NO release which lead to vasodilation, cellular proliferation and survival, matrix metalloproteinase synthesis, and release of proangiogenic factors, IL-6, IL-8, and VEGF. These factors enhance melanoma development and progression [270]. β-Adrenoceptor antagonists such as propranolol are now known to exhibit antiproliferative properties with good safety profiles. Propranolol reverses the downstream cancer progression effects and stimulates cell apoptosis [271–273]. As such, β-blockers (e.g., propranolol, nebivolol, carvedilol, and labetalol) have therapeutic potential for the treatment of intraocular tumors. Some β-blockers have already gone through trials and have been approved by the Food and Drug Administration [274, 275]. According to Farhoumand et al. [276], nebivolol exclusively showed antitumor activity in uveal melanoma among all β-blockers in their investigation.

### 5.6. Targeting the β-Adrenoceptor in Cancer Therapy

Cancer is one of the leading causes of patient morbidity and mortality worldwide. Several cancer therapies have been developed, yet without the much needed efficiency, efficacy, and/or safety profiles. Currently, research has geared toward understanding the role of the sympathetic system in cancer progression via β-adrenoceptor activation by catecholamines [277]. This intriguing school of thought has been extensively researched and reviewed in detail by several scientists [278–281]. It had already been established in various *in vitro* and *in vivo* studies that the activation of β-adrenoceptors by catecholamines promotes the synthesis of cytokines especially IL-6 [282], cancer cell immunity [283], initiation of tumorigenesis [284], stimulation of tumor-associated macrophage [285], VEGF/FGF2-mediated angiogenesis [286], potentiation of the tumor micro- and macroenvironments [287, 288], and cancer cell proliferation, differentiation, and migration in animal models of various cancers [278, 289–291]. β-Adrenoceptor activation can stimulate activation of mitogen-activated protein kinase (MAPK) family of proteins via ERK. Both MAPK and ERK phosphorylate nuclear transcription factors to regulate expression of multiple genes involved in cell proliferation [292]. These characteristics of β-adrenoceptor activation tend to enhance cancer cell progressivity.

β-Adrenoceptor activation is reported to also regulate cellular metabolism which in turn promotes cancer cell progressivity, cell inflammation, cell apoptosis [293], cell communication and movement, DNA damage repair, etc. [294]. This implies that blocking β-adrenoceptor activation with β-blockers, in principle, will downregulate cancer progressivity. For these reasons, all 3 β-adrenoceptor subtypes are being targeted for treatment of various cancers [295]. β-Blockers (e.g., nebivolol, carvedilol, and propranolol) are inexpensive. They have good safety profiles, prevent tissue exposure to hazardous radiations employed in radiotherapy, and enhance sensitivity to chemotherapy agents such as response to anti-PD-1 and anti-CTLA4 [296–298]. They antagonize the effect of catecholamines yielding potentially beneficial outcomes. Indeed β-adrenoceptor antagonism has already been employed and has shown promising clinical effects in breast cancer. Propranolol is currently the gold standard in infantile hemangioma treatment [259]. Propranolol has also shown antitumor effects on colorectal and breast cancers, glioblastoma, ovarian carcinoma, and pancreatic cancers [299–301].

### 5.7. Targeting the β_3_-Adrenoceptor in Other Therapies

Research is still ongoing to unravel more novel therapeutic potentials of the β_3_-adrenoceptor. The list is not exhaustive. For example, nonalcoholic fatty liver disease (NAFLD) is a liver condition characterized by lipid accumulation in hepatocytes and hepatocyte steatosis. With rapid development and as economies improve, more and more people are currently consuming less vegetables and more meat. Fatty meat, animal visceral food, fried foods, and sweets are rich in saturated fatty acids and cholesterol. Sugars are normally stored as glycogen but can also be converted to fats for storage. Moreover, people are infatuated with technology rather than performing physical tasks which result in less exercise and high build-up of fat stores ultimately leading to an increase in the prevalence of NAFLD [136]. NAFLD is in turn a risk factor for other disease conditions including type 2 diabetes, dyslipidemia, hypertension, and cardiovascular diseases. Wang et al. [302] recently tested the hypothesis that β_3_-adrenoceptor expression is upregulated in NAFLD, and that BRL 37344 (a β_3_-adrenoceptor selective agonist) treatment is protective against liver steatosis and inflammation. Wang and colleagues employed a male Sprague Dawley rat model of a HFD-induced NAFLD. Setting up 4 groups of experiment rats, control (fed with SD), HFD (fed with HFD), HFD + BRL 37344, and HFD + L748337 (a β_3_-adrenoceptor antagonist), they showed that levels of ALT, AST, triglycerides, total cholesterol, low density lipoprotein cholesterol, and FFA levels relatively decreased among the group that had been treated with the β_3_-adrenoceptor agonist (BRL37344). Additionally, BRL 37344-treated rats had lower body and liver weights, liver index values, and lipid droplet accumulation compared to controls. Besides, reduction in NAFLD activity scores (NASs) also suggested liver steatosis and inflammation were ameliorated in BRL37344-treated rats. However, these parameters were reversed in L748337-treated rats compared to controls. In addition, while β_3_-adrenoceptor, PPAR-*α* (peroxisome proliferator-activated receptor alpha), and mCPT-1 (mouse cell protein 1) expressions were upregulated by treatment with BRL 37344, PPAR-*γ* (peroxisome proliferator-activated receptor gamma) and FAT/CD36 (fatty acid translocase) expressions were downregulated. Decrease in FAT/CD36 expression reduced entry of fatty acids into liver, whereas increase in mCPT-1 facilitated transport of hepatocellular fatty acids into mitochondria to be metabolized. Increase in PPAR-α (a transcription factor) expression decreased hepatocyte apolipoprotein C-III expression and increased lipoprotein lipase gene expression in order to enhance triglyceride metabolism. PPAR*γ* is a transcription factor that downregulates immune cell function by increasing expression of anti-inflammatory related gene expression and downregulates expression of proinflammatory mediators which essentially reduce inflammation. Their results therefore indicate that upregulation of β_3_-adrenoceptor overexpression coupled with β_3_-adrenoceptor-G_s_ signal overactivity is protective against liver steatosis and inflammation and is suggestive of appreciable potential of BRL 37344 as a therapeutic target for the treatment of NAFLD [302].

Alzheimer's disease is a condition characterized by a decline in cognitive ability, gradual neurodegeneration, and the development of amyloid β (Aβ)-plaques and neurofibrillary tangles constituting hyperphosphorylated tau [303]. To date, there is no curative treatment for the disease. Alzheimer's disease is associated with defects in thermoregulation and cold-induced tau hyperphosphorylation in mammals and so β_3_-adrenoceptor activation may be beneficial in the treatment of Alzheimer's disease. Tournissac et al. [304] assessed this hypothesis and have reported experimental evidence in a triple transgenic mouse model that administration of a specific β_3_-adrenoceptor agonist (CL 316243) decreases body weight and improves peripheral glucose metabolism as well as brown adipose tissue thermogenesis in concordance with earlier studies [305]. More importantly, they stated that treated transgenic mice improved in recognition index by 19% while locomotion, anxiety, and tau pathology were unaffected. Besides, insoluble Aβ42/Aβ40 ratio also decreased by 27% in the hippocampus of CL 316243-treated mice. All together, these experimental outcomes suggest that CL 316243 might have significant potential for the synergistic treatment of Alzheimer's disease including some metabolic syndromes such as type 2 diabetes [304].

High level of cAMP is believed to be involved in the pathogenesis of autosomal dominant polycystic kidney disease (ADPKD) which is a condition characterized by progressive expansion of fluid-filled cysts from various nephron segments that disrupt the parenchyma. It is now known that increased intracellular cAMP concentration in the cyst epithelial cells promotes cell proliferation as well as fluid secretion which results in cyst expansion [306]. To date, only vasopressin type 2 receptor blockade with tolvaptan remains the only FDA-approved remedy [307]. Vasopressin type 2 receptor activation results in elevated cellular cAMP levels. Another pathway leading to an increase in cAMP levels is the activated β_3_-adrenoceptor–induced cAMP synthesis by AC. β_3_-Adrenoceptor is expressed in human renal cyst-lining epithelial cells. Besides, β_3_-adrenoceptor expression is upregulated in ADPKD mice. As such, β_3_-adrenoceptor blockade may be a novel approach to treating ADPKD. Schena et al. [61] demonstrated that administration of SR59230A (a β_3_-adrenoceptor selective antagonist) to an ADPKD mouse model ameliorates cAMP levels with concomitant reduction in kidney/body weight ratio and a significant improvement in kidney function. SR59230A therefore has potential to be developed for ADPKD therapy.

More recent data unravel the potential of β_3_-adrenoceptor in the management of preterm birth which is the leading cause of infant morbidity and mortality. β_3_-Adrenoceptor is expressed in uterine myocytes and microvascular endothelial cells of women. Its expression is upregulated in myometrium of pregnant women suggesting a role of this receptor in pregnancy [278]. Asif and colleagues [278] observed that mirabegron (a selective β_3_-subtype agonist) in the tissue both relaxed oxytocin-induced contraction of the myometrium with an EC_50_ of 41.5 *μ*M. Their data taken together give experimental evidence that underscores the potential of mirabegron or as part of a combination with others as a uterine tocolytic. β_3_-Adrenoceptor is thought to relax myometrium in a cGMP-independent manner. However, more research is required to fully understand the mechanisms underlying the β_3_-adrenoceptor stimulation of myometrium relaxation.

## 6. Conclusion and Perspectives

β-Adrenoceptor signaling cascades regulate crucial physiological processes involved in multiple pathogenesis and therefore remain potentially vital for the development of therapeutics. The strategies discussed for possible development of therapeutics here are but a few. More characterization reports unraveling novel modalities, new biochemical pathways, and physiological roles of β_3_-adrenoceptor are rapidly being revealed which make the β_3_-adrenoceptor an intriguing target for drug discovery. Most studies are still at the preliminary stages, for instance, the proposed use of β_3_-adrenoceptor in the treatment of Alzheimer's disease, ADPKD, and preterm birth. This notwithstanding, the role of β-adrenoceptors, generally, in some tissues remains ambiguous. Many questions remain unanswered and clinical trials versus sight effects have not been assessed as most hypotheses have only been tested at the level of disease animal models. As β-adrenoceptor activation or inhibition may couple multiple downstream signal molecules, can a particular disease be targeted without necessarily activating redundant signals? While some disease pathologies cannot be duplicated by animal models, some findings between scientists have been controversial and some clinical trials for the few at advanced stages have been disappointing. However, taken together, the development of β-adrenoceptor agonists and antagonists for novel therapeutic approaches is far more promising than ever as some have been approved for use by the FDA while others have reached advanced stages in clinical trials—Phase II and III. Meanwhile, research is rapidly revealing new pharmacological approaches. Developing new strategies requires understanding of the underlying molecular and biochemical basis of disease pathology. The β_3_-adrenoceptor in particular has suffered a setback owing to its late discovery, a few controversial, inconsistent, and/or contradictory findings, and the lack of selective detection tools coupled with interspecies differences. To bridge controversial findings, more insights into the structure of the β_3_-adrenoceptor, its function, regulation, expression patterns, roles in disease pathology, etc. will enhance the drug discovery process. To do so, more appropriate disease animal models and techniques are required in order to fully explore the potential of the β_3_-adrenoceptor as well as the β_1_- and β_2_-subtypes to fast-forward the developmental processes. Should the therapeutic potentials of the β-adrenoceptors be fully explored, the healthcare and lives of many patients especially those with chronic issues will be transformed.

## Figures and Tables

**Table 1:** Human major tissue distribution and physiological functions of adrenoceptor subtypes.

AR subtype	Tissue distribution	Physiological function
α_1A_	Cerebral cortex, cerebellum, heart, liver, prostate, lymphocytes	Contraction of urethral smooth muscle, contraction of arterial smooth muscle
α_1B_	Spleen, kidney, endothelial cells, heart osteoblasts, lymphocytes, somatic veins, and arteries	Contraction of arteries/veins, osteoblastic cell proliferation, promotes cardiac remodeling
α_1D_	Cerebral cortex, carotid artery, aorta, lymphocytes, heart, prostate, blood, vessels, bladder, platelets	Ureteral muscle contraction, contraction of arteries, vasoconstriction of aorta and coronary artery
α_2A_	Brain, kidney, liver, heart, spleen, aorta, lung, skeletal muscle, platelets sympathetic neurons, pancreas	Inhibition of presynaptic norepinephrine release, vasoconstriction of vessels in skeletal muscle, involved in baroreceptor reflex, sedation, hypotension, analgesic
α_2B_	Brain, kidney, liver, heart, lung, aorta, skeletal muscle, spleen	Vasoconstriction
α_2C_	Brain, kidney, liver, heart, spleen, aorta, lung, skeletal muscle, platelets, pancreas, coronary and CNS vessels, sympathetic neurons	Inhibition of presynaptic norepinephrine release, analgesic, sedation
β_1_	Brain, lungs, liver, kidney, spleen, skin, adipose tissue, lymphocytes, coronary arteries, intestinal muscle	Increase heart rate (conduction, contractility, automaticity), renin release, lipolysis, vasodilation, relaxation of intestinal muscle
β_2_	Brain, lungs, liver, lymphocytes, skin, heart, eye, vascular smooth muscle, uterus, bladder, adipocytes, sympathetic terminal	Vasodilation, bronchodilation, bladder and uterus relaxation, lipolysis, glycogenolysis, positive ionotropic/chronotropic, norepinephrine release
β_3_	Adipose tissue, brown tissue, gut, urinary bladder, gall bladder, eye, small intestine	Thermogenesis, lipolysis, myometrium relaxation, vasodilation of coronary arteries, negative ionotropic effect

Abbreviations: AR, adrenoceptor; CNS: central nervous system.

**Table 2:** Characteristic differences between human *β*-adrenoceptors.

Property	Receptor subtype		
	*β*_1_	*β*_2_	*β*_3_
No. of amino acids	477	413	408
No. of introns	—	—	1
No. of exons	1	1	2
Phosphorylation by PKA and *β*-ARK	Yes	Yes	No
Effector enzyme	AC	AC	AC, NO synthase
G protein type	G_*s*_	G_*s*_ or G_*i*_	G_*s*_ or G_*i*_
Sequence homology	54% with *β*_2_	46% with *β*_3_	51% with *β*_1_

Abbreviations: AC, adenylyl cyclase; NO, nitric oxide.

## Data Availability

The data used to support the findings of this study are included within the article and are available upon request from the corresponding author.

## Nomenclature

β-ARK(s): β-adrenergic kinase(s)
AC: Adenylyl cyclase
ADP: Adenosine diphosphate
Anti-PD-1: Anti-programmed cell death protein 1
ATF-2: Activating transcription factor 2
ATP: Adenosine triphosphate
CaMKII: Ca^2+^/calmodulin-dependent protein kinase II
cAMP: Cyclic adenosine monophosphate
CBP: CREB-binding Protein
CEBP*α*: CCAAT enhancer binding protein alpha
CGI-58: Comparative gene identification-58
cGMP: Cyclic guanosine monophosphate
COP-1: Constitutive photomorphogenesis protein 1
CRE: cAMP response element
CREB: CREB-binding element
DAG: Diacyl glycerol
EGFR: Epidermal growth factor receptor
eNOS: Nitric oxide synthase
EPAC: Exchange protein activated by cAMP
ERK: Extracellular regulated kinase
FAT/CD36: Fatty acid translocase/platelet glycoprotein 4
FFA(s): Free fatty acid(s)
GC: Guanylyl cyclase
GDP: Guanosine diphosphate
GPCR(s): G protein-coupled receptor(s)
G protein: Guanine nucleotide-binding protein
GRK2: G protein linked receptor kinase 2
GRKs: G protein linked receptor kinases
GTP: Guanosine triphosphate
HFD: High-fat diet
HSL: Hormone-sensitive lipase
IL-6: Interleukin 6
IL-8: Interleukin 8
IP_3_: Inositol 1,4,5-triphosphate
L-TCC: L-type calcium channel
MAPK: Mitogen-activated protein kinase
MaxiK: Large-conductance Ca^2+^-activated K^+^
NMDA: N-methyl-D-aspartate
NO: Nitric oxide
PPAR-α: Peroxisome proliferator-activated receptor alpha
PPAR-γ: Peroxisome proliferator-activated receptor gamma
PIP_2_: Phosphatidylinositol 4,5-bisphosphate
PI-PLC: Phosphoinositide-phospholipase C
PKA: cAMP-dependent protein kinase A
PKC: Protein kinase C
PKG: Protein kinase G
PLB: Phospholamban
PLC: Phospholipase C
PLIN: Perilipin
RAP2A: Ras-related protein Rap-2a
RyR: Ryanodine receptor
SD: *Standard diet*
SMAD: *Suppressor of Mothers against Decapentaplegi*
SNS: Sympathetic nervous system
TM(s): Transmembrane(s)
TNFR: Tumor necrosis factor alpha receptor
TNF-α: Tumor necrosis factor alpha
TRIB1: Tribbles pseudokinase 1
T-TCC: T-type calcium channel
Ub: Ubiquitin
UCP1: Uncoupling protein 1
VEGF: Vascular endothelial growth factor
